# Diagnostic value of aberrant decreased 5-Methylcytosine RNA modification in leukocytes for non-small cell lung cancer

**DOI:** 10.7150/jca.85681

**Published:** 2023-07-16

**Authors:** Mao Huang, Liang Ming, Hongbo Jiang, Ping Jiang, Xi Jiang, Haofan Yin, Honghai Hong

**Affiliations:** 1 Department of Clinical Laboratory, Zhuhai People's Hospital (Zhuhai Hospital affiliated with Jinan University), Zhuhai, Guangdong, China.; 2 Department of Clinical Laboratory, The Third Affiliated Hospital of Guangzhou Medical University, Guangzhou, Guangdong, China.; 3 Department of Medical Laboratory, Shenzhen People's Hospital (The Second Clinical Medical College, Jinan University; The First Affiliated Hospital, Southern University of Science and Technology), Shenzhen, Guangdong, China.; 4 Department of Clinical Medical Laboratory, Guangzhou First' People Hospital, School of Medicine, South China University of Technology, Guangzhou, Guangdong, China.; 5 Department of Clinical Laboratory, The Seventh Affiliated Hospital of Sun Yat-sen University, Shenzhen, Guangdong, China.

**Keywords:** 5-Methylcytosine, non-small cell lung cancer, leukocytes, diagnosis, biomarker

## Abstract

**Background:** Non-small cell lung cancer (NSCLC) was a disease with poor outcomes, partly because there were no high-efficiency non-invasive diagnostic biomarkers. The RNA modification status of 5-Methylcytosine (m^5^C) has been shown to be a biomarker for various diseases, but its potentiality to be a diagnostic biomarker for NSCLC remained inconclusive.

**Methods:** In this research, we collected peripheral leukocyte samples from 141 patients with NSCLC and 90 normal people as controls to evaluate the extent of m^5^C RNA modification.

**Results:** We found that the m^5^C modification levels in leukocytes of NSCLC patients were decreased dramatically, which were compared to the normal controls, and levels of m^5^C modification decreased progressively with tumor stage. Importantly, m^5^C modification exhibited superior diagnostic value compared to carcinoembryonic antigen (CEA), squamous cell carcinoma antigen (SCC), cytokeratin 19 fragment (Cyfra21-1), and carbohydrate antigen 125 (CA125), which demonstrated area under the curves (AUCs) of 0.912, 0.773, 0.669, 0.754, and 0.732, respectively. The combination of m^5^C modification with these serum tumor biomarkers further improved the AUC to 0.960. A nomogram model incorporating m^5^C modification also provided an effectively diagnostic tool for NSCLC.

**Conclusion:** Collectively, our findings suggested that m^5^C modification in leukocytes held promise as a prospective biomarker for NSCLC diagnosis.

## Introduction

According to current statistics, lung cancer is the most prevalent tumor worldwide, accounting for the highest incidence and mortality rate of all malignancies in men and the second highest in women^1^. NSCLC was the main categories of lung cancer, and it remained a huge challenge in clinical management. Early detection of NSCLC was crucial for improving the five-year survival rates following surgical intervention. Unfortunately, the absence of early clinical symptoms and diagnostic techniques resulted in over 70% of NSCLC cases being identified at advanced stages^2^. Therefore, it was crucial to diagnose NSCLC accurately and at an early stage to improve patient prognosis.

Due to limited clinical resources, large-scale imaging screening and invasive pathology were currently not feasible for population-based screening of NSCLC^3^. As a result, serological tests had become increasingly popular for non-invasive and dynamic clinical surveillance of NSCLC patients^4^. However, currently available clinical diagnostic biomarkers, such as SCC, CEA, CA125, and Cyfra21-1, exhibited inadequate sensitivity and specificity for NSCLC screening, particularly in early-stage patients^5^-^7^. Consequently, a non-invasive and efficient method to improve the early diagnosis of NSCLC was urgently needed.

RNA methylation modifications, which included 5-Methylcytosine (m^5^C) and N6-methyladenosine (m^6^A), were regulated by a family of Writers, Erasers and Readers^8^, ^9^. These modifications were primarily concentrated within the CDS and 3'UTR regions of RNA, and could affect RNA transcriptional, post-transcriptional, and translational processes, making them a key regulator of various diseases^9^, ^10^. Our previous research, as well as other studies, had shown that m^6^A modification levels in leukocytes were markedly increased in NSCLC patients and closely associated with the key Erasers molecules FTO and ALKBH5, which is suggested that m^6^A methylation can be used as a tumor biomarker to modify the peripheral blood leukocytes^11^, ^12^. Additionally, our previous study has identified m^5^C methylation modification levels of white bold cells in colorectal cancer (CRC) patients as a novel tumor marker, with elevated levels seen in various primary CRC mouse models^13^. Therefore, we aimed to investigate whether m^5^C modification status in leukocytes could be a novel tumor biomarker in diagnosing NSCLC.

Surprisingly, our research found that, in contrast to CRC patients, the level of m^5^C RNA modification detected in leukocytes of NSCLC patients was apparently reduced. Furthermore, m^5^C modification provided exceptional diagnostic capability for NSCLC patients. The combination of m^5^C modification with other serum tumor biomarkers and the construction of a nomogram model incorporating m^5^C modification could further enhance diagnostic efficacy.

## Materials and Methods

### Human samples

The Institutional Review Board of The Third Affiliated Hospital of Guangzhou Medical University had approved the retrospective study (IRB number: S2021-114) on 20 Jan 2021. Between Jan 2021 and Dec 2022, we collected 231 peripheral blood samples using EDTA anticoagulation tubes at The Third Affiliated Hospital of Guangzhou Medical University. Blood samples were taken to test for tumor markers including SCC, CA125, CEA and Cyfra21-1 in the clinical laboratory. After the necessary tests, any remaining blood was collected with the patient's consent. Among those samples, 141 samples were from NSCLC patients and the other 90 samples were normal controls (NC) without a history of diabetes, hypertension, stroke, aneurysm, myocardial infarction or other serious diseases. 0.5 mL whole blood were mixed with 1 mL red blood cell lysis solution (TIANGEN, Beijing, China), after centrifuged, 1 mL triol was used to dissolve the precipitate to stabilize RNA. Then put the mixed samples at -80℃ to store. All the patients with NSCLC were selected according to the result of histopathology or cytopathology, and informed consent was obtained from all participants. 141 NSCLC patients were followed and had the blood samples both at time of first diagnosis before they got treatment (including surgery, chemotherapy, radiotherapy, targeted therapy, or immunotherapy) and samples from 11 NSCLC patients were collected at six months after they underwent therapy. Ethics approval was obtained from the Ethics Committee of The Third Affiliated Hospital of Guangzhou Medical University. All the patients' clinical and biological characteristics are displayed in Supplementary Table 1 and Supplementary Table 2.

### RNA isolation and m^5^C quantification

TRIzol (Thermo Scientific, MA, USA) was used to extract the RNA. Levels of m^5^C in RNA were quantified using the MethyFlash 5-mC RNA Methylation ELISA Easy Kit (Fluorometric) (Epigentek, New York, USA). Briefly, added 200ng RNA into the assay wells which were coated with biding solution, and then incubated at 37℃ for 90minutes.

Subsequently, a serial diluted concentration of 5-mC antibody, signal indicator and enhancer solution were added consecutively, then incubated at room temperature for 1 hour. Finally, added fluorescent developer and incubated for another 3 minutes at room temperature. Using synergyH1 multi-mode readers (BioTek, Vermont, USA) to measure the fluorescence at wavelengths of 530 nm for excitation and 590 nm for emission, within 2 to 10 minutes after adding the development solution.

### Statistical analysis

For normally distributed data between two groups, unpaired Student's t test was used to analyze the variability, which was shown as the SD (mean±SD). Otherwise, nonparametric Mann-Whitney test was used to analyze the data. For multiple groups, the significant differences were determined by one-way analysis of variance (ANOVA).

There was a statistical significance if P was less than 0.05. The receiver operating characteristic (ROC) curve with an area under the curve (AUC) was used to assess the diagnostic value of biomarkers to differentiate NSCLC from NC. A nomogram was constructed based on multivariate logistic regression analysis for predicting NSCLC.

## Results

### Levels of m^5^C modification in leukocytes of NSCLC patients

**Figure 1:**
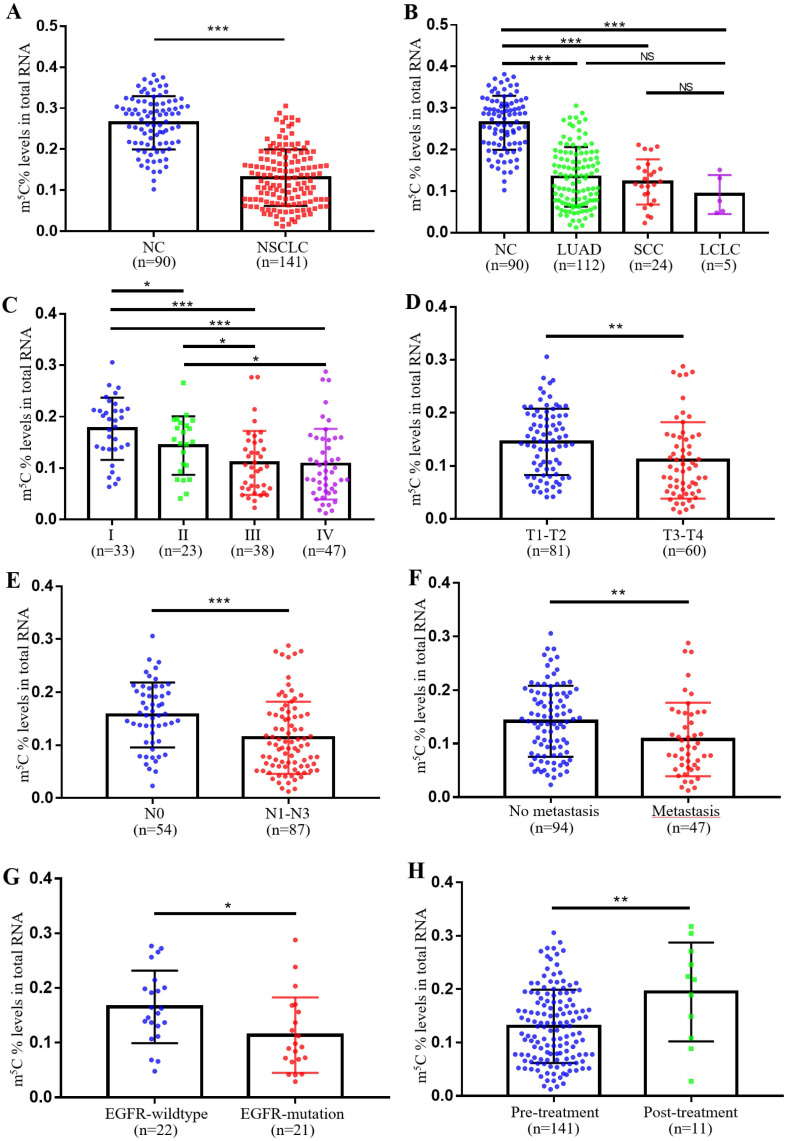
** Levels of m^5^C modification in leukocytes of NSCLC patients.** (A) Levels of m^5^C modification in RNA of peripheral blood leukocytes from NC (n=90) and NSCLC patients (n=141). (B) m^5^C modification levels of leukocytes in different pathological classifications of NSCLC, including LUAD (adenocarcinoma, n=112), SCC (squamous cell carcinoma, n=24), and LCLC (large cell lung carcinoma, n=5). (C) m^5^C modification levels of leukocytes in NSCLC patients at different clinical stages, including stage-I (n=33), stage-II (n=23), stage-III (n=38), and stage-IV (n=47). (D) Comparison of m^5^C modification levels of leukocytes in NSCLC patients with different T stages. (E) Comparison of m^5^C modification levels of leukocytes in NSCLC patients with different N stages. (F) Comparison of m^5^C modification levels of leukocytes in NSCLC patients with (n=47) and without (n=94) distant-metastasis. (G) Comparison of m^5^C modification levels of leukocytes in NSCLC patients with (n=21) and without (n=22) EGFR mutation. (H) Comparison of m^5^C modification levels of leukocytes in NSCLC patients with (n=11) and without (n=141) treatment. Data were shown as mean ± SD; * *P* <0.05, ** *P* <0.01, and *** *P* <0.001. NS, no significance.

In our previous study, we observed remarkably higher levels of m^5^C RNA modification in leukocytes of CRC patients. Therefore, we further checked its expression in leukocytes of NSCLC patients. Supplementary Table 1 showed the baseline characteristics of NC individuals and NSCLC patients were well-matched. Surprisingly, unlike in CRC, NSCLC patients exhibited markedly lower levels of m^5^C modification in their leukocytes (Fig. 1A). Furthermore, no significant difference in m^5^C modification was found between different pathological classifications of NSCLC, which included adenocarcinoma, squamous cell carcinoma, and large cell lung carcinoma (Fig. 1B). Statistical analyses presented that the m^5^C levels were associated with gender, clinical stage, N classification, M classification, differentiation, and EGFR genotyping (Supplementary Table 2). Among them, levels of m^5^C RNA modification gradually decreased with tumor stage progression (Fig. 1C). As T classification and N classification increased, levels of m^5^C RNA modification were also reduced in leukocytes (Fig. 1D-E). NSCLC patients with distant-metastases had lower m^5^C levels than non-metastatic NSCLC patients (Fig. 1F). The results also indicated that poor prognosis NSCLC patients with EGFR mutations shared reduced m^5^C levels (Fig. 1G). Additionally, Leukocyte m^5^C levels rebounded in NSCLC patients after treatment (Fig. 1H). Taken together, our findings demonstrated that in contrast to CRC patients, levels of m^5^C RNA modification detected in leukocytes of NSCLC patients decreased apparently.

### Comparison of m^5^C modification levels in leukocytes and tumor biomarker levels in serum

**Figure 2:**
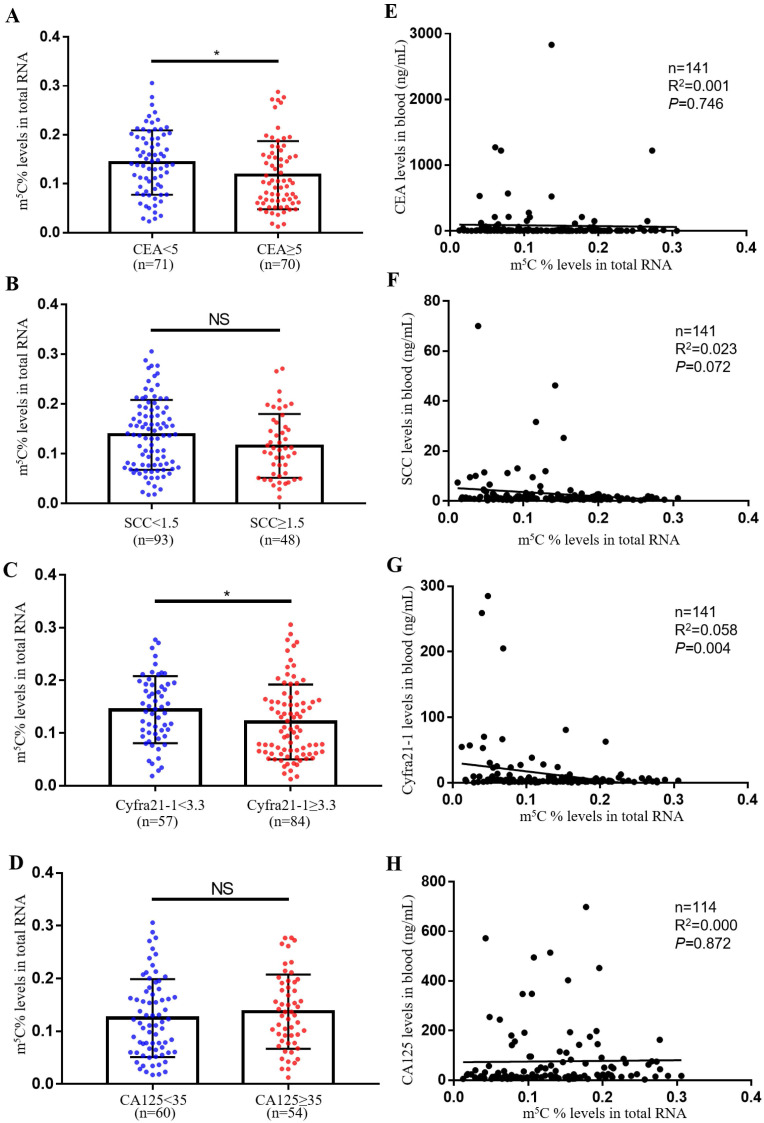
** Comparison of m^5^C modification levels in leukocytes and tumor biomarker levels in serum.** (A) Comparison of m^5^C modification levels of leukocytes in NSCLC patients with CEA ≥ 5 ng/ml (n = 70) and CEA < 5 ng/ml (n = 71). (B) Comparison of m^5^C modification levels in leukocytes between NSCLC patients with SCC≥ 1.5 ng/ml (n = 48) and those with SCC < 1.5 ng/ml (n = 93). (C) Comparison of m^5^C modification levels in leukocytes between NSCLC patients with Cyfra21-1≥ 3.3 ng/ml (n = 84) and those with Cyfra21-1< 3.3 ng/ml (n = 57). (D) Comparison of m^5^C modification levels in leukocytes between NSCLC patients with CA125≥ 35 ng/ml (n = 54) and those with CA125< 35 ng/ml (n = 60). (E-H) Spearman correlation analysis of m^5^C modification levels with CEA levels (E), SCC levels (F), Cyfra21-1 levels (G), and CA125 levels (H) in NSCLC patients. Data were shown as mean ± SD; * *P* <0.05. NS, no significance.

The most usually used serum biomarkers for NSCLC were SCC, CA125, CEA and Cyfra21-1. In this study, we investigated the relationship between the level of m^5^C modification and these tumor biomarkers. Notably, we observed that m^5^C modification levels were lower in the CEA and Cyfra21-1 abnormally increased groups compared to the control group (Fig. 2A-D). However, we only found a negative correlation between m^5^C modification levels and Cyfra21-1 levels, and no significant correlation with CEA, SCC, and CA125 levels (Fig. 2E-H). Therefore, Cyfra21-1 was identified as the serum biomarkers of NSCLC that was most associated with m^5^C modification of leukocytes.

### Clinical utility of m^5^C modification levels in leukocytes for the diagnosis of NSCLC

**Figure 3:**
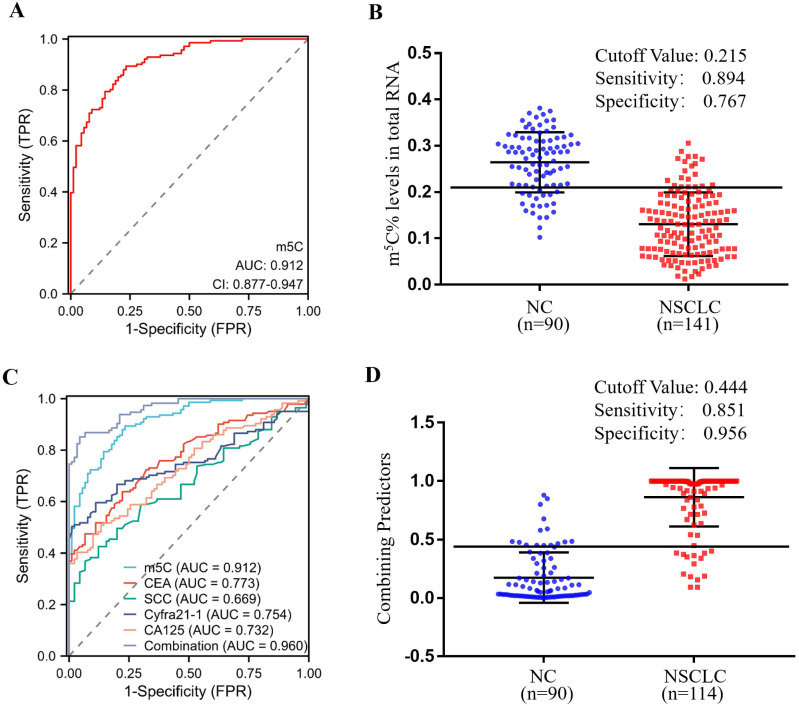
** Clinical utility of m^5^C modification levels in leukocytes for the diagnosis of NSCLC.** (A, B) ROC curve (A) and corresponding cutoff value (B) for discriminating NSCLC from NC based on m^5^C modification levels in leukocytes. (C) ROC curve for m^5^C modification, CEA, SCC, Cyfra21-1, and CA125 alone or together. (D) Cutoff value for m^5^C modification, CEA, SCC, Cyfra21-1, and CA125 in combination for discriminated NSCLC from NC.

In order to estimate the diagnostic value of m^5^C modification in leukocytes for NSCLC furtherly, ROC curves were generated. As illustrated in Fig. 3A, m^5^C modification displayed a high ability to distinguish NSCLC patients from NC, with an AUC of 0.912 (95% CI 0.877 to 0.947). The optimal cutoff value for m^5^C modification level, based on the Youden index, was determined to be 0.215, with a sensitivity of 0.894 and specificity of 0.767 (Fig. 3B). Moreover, our findings suggested that m^5^C modification possessed superior diagnostic value compared to CEA, SCC, Cyfra21-1, and CA125, which demonstrated AUCs of 0.773, 0.669, 0.754, and 0.732, respectively (Fig. 3C; Table 1). While m^5^C modification was combined with these commonly used serum biomarkers, the AUC of the biomarker panel increased to 0.960 (Fig. 3C). The combined biomarker score for diagnosis was defined as: 0.539-23.816*m5C+0.084*CEA+0.682*SCC+0.701*Cyfra21-1+0.060*CA125. The optimal cutoff value for the combination biomarker was 0.444, with a sensitivity of 0.851 and specificity of 0.956 (Fig. 3D). Taken together, these results indicated that m^5^C modification levels in leukocytes RNA provided exceptional diagnostic capability for NSCLC patients.

### The diagnostic value of m^5^C modification levels in leukocytes at different stages of NSCLC

**Figure 4:**
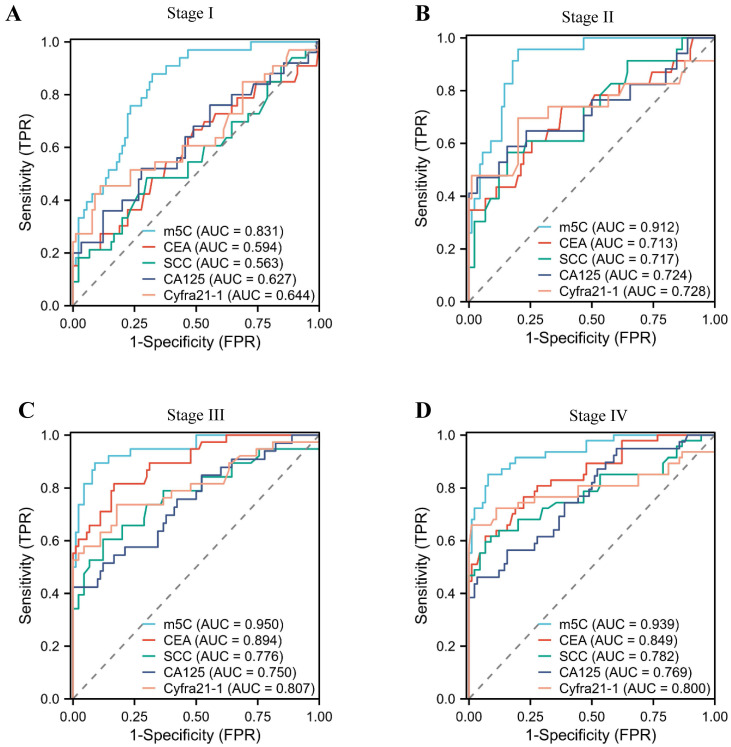
** The diagnostic value of m^5^C modification levels in leukocytes at different stages of NSCLC.** (A-D) ROC curve for m^5^C modification compared with CEA, SCC, Cyfra21-1, and CA125 in NSCLC patients at stage I (A), stage II (B), stage III (C), and stage IV (D).

In addition, we assessed the diagnostic potential of m^5^C modification in leukocytes at various stages of NSCLC. Notably, stage I NSCLC patients could be accurately distinguished from healthy individuals by m^5^C modification, with AUCs of 0.831 (Fig. 4A). Conversely, conventional used serum biomarkers provided limited diagnostic capability for stage I NSCLC patients (Fig. 4A; Supplementary Table 3). Encouragingly, the AUC of m^5^C modification for NSCLC patients increased gradually with advancing stage (Fig. 4B-D). These results underscored the potential of m^5^C modification in leukocytes as a promising diagnostic biomarker for early NSCLC.

### A nomogram for predicting the probability of NSCLC for healthy individuals

**Figure 5:**
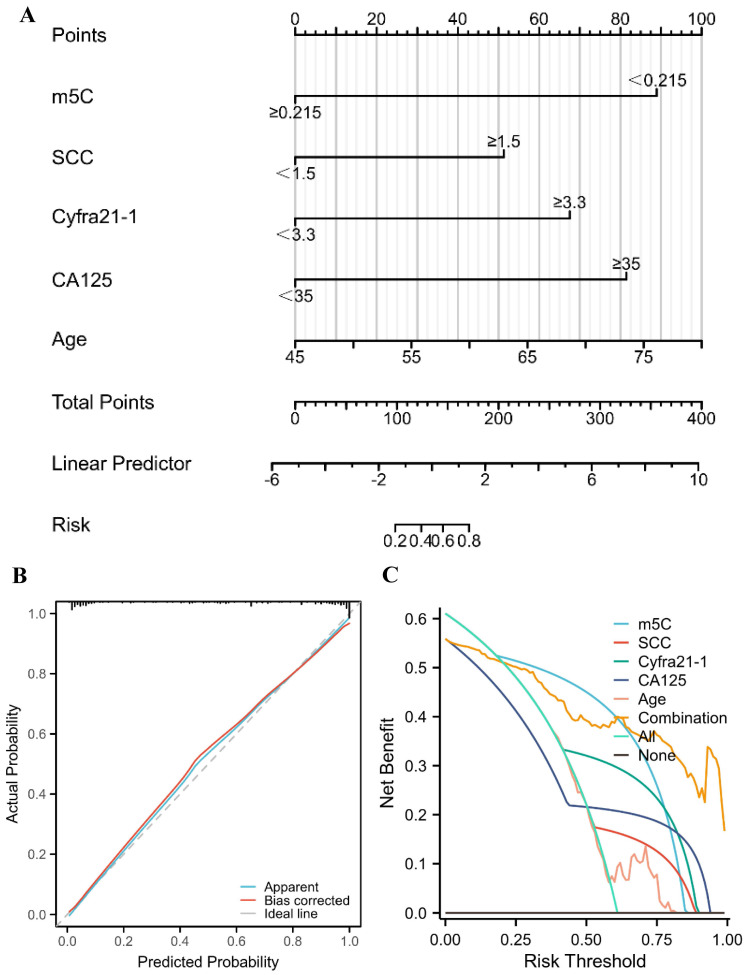
** A nomogram for predicting the probability of NSCLC for healthy individuals.** (A) A nomogram for differentiating between NSCLC patient and healthy individual was established based on multivariate logistic regression results. (B) Calibration curves of the nomogram. (C) DCA for the nomogram and various markers alone.

The risk of NSCLC that related to m^5^C modification, CEA, SCC, Cyfra21-1, CA125, and age was indicated by the results of univariate logistic regression analysis (Table 2). Furthermore, m^5^C modification, SCC, Cyfra21-1, CA125, and age were independent diagnostic factors for NSCLC, which were identified by multivariate logistic regression analyses (Table 2). According to the results of the multivariate logistic regression, a nomogram was constructed to differentiate NSCLC patients from healthy individuals (Fig. 5A). The concordance index (C-index) of the nomogram was 0.945 (95% CI 0.917 to 0.972), suggesting a strong capacity to distinguish NSCLC patients from healthy individuals (Fig. 5A). The calibration curves of the nomogram displayed high consistencies between observed and predicted values, with the Hosmer-Lemeshow test yielding a non-significant P value of 0.361 (Fig. 5B). Furthermore, the clinical validity of the nomogram model was confirmed by the decision curve analysis (DCA), which obtained a net benefit for almost all threshold probabilities (Fig. 5C). Collectively, a nomogram model incorporating m^5^C modification also provided an effectively diagnostic tool for NSCLC.

## Discussion

Growing evidence suggested that m^5^C RNA modification and its regulators were abnormally expressed in different types of cancers^14^. Meanwhile, m^5^C RNA methylation status was closely linked to cancer pathogenesis, including cancer development, metastasis, recurrence, and drug resistance^15^. Previous study had identified abundant m^5^C modification in the lncRNA of CRC, which participated in the transcription of lncRNA by influencing the promoters and super-enhancers^16^. our recent study also found significantly increased levels of m^5^C RNA modification in leukocytes from CRC patients as a novel tumor biomarker^13^. Given the lack of effective non-invasive diagnostic biomarkers for NSCLC, we proposed to investigate whether the m^5^C modification status in leukocytes could also be used as a tumor biomarker for NSCLC.

The relationship between m^5^C modification and NSCLC has been controversial^15^. One of the earliest studies found that elevated levels of m^5^C modification could be detected in circulating tumor cells from NSCLC patients^17^. While recent study also demonstrated that NSUN3 and NSUN4, regulators of m^5^C modification, could promote NSCLC progression^18^. Meanwhile, NSUN3 was strictly linked to the infiltration of CD8^+^ T cells, whereas NSUN4 was associated with the infiltration of neutrophils^18^. However, in contradiction to these results, m^5^C methylation modification patterns were constructed by bioinformatic methods based on 11 m^5^C regulators, with higher scores suggesting a better prognosis^19^. In our study, the level of m^5^C modification of leukocytes in NSCLC patients was markedly lower than that in NC (Fig. 1), although the reasons for this reduction and the specific regulators that played a crucial role required further investigation. The pathological subtype of large cell lung cancer, which was associated with a poorer prognosis, exhibited relatively lower levels of m5C compared to the pathological subtypes of lung squamous carcinoma and lung adenocarcinoma, which were associated with a better prognosis. However, this difference did not reach statistical significance, likely due to the limited number of cases of large cell lung cancer in our study (Fig. 1B; Supplementary Table 2).

Nevertheless, abnormally reduced m^5^C modification exhibited superior diagnostic value compared to CEA, SCC, Cyfra21-1, and CA125, which demonstrated AUCs of 0.912, 0.773, 0.669, 0.754, and 0.732, respectively (Fig. 3A-B). The combined biomarker score of m^5^C modification in combination with these traditional biomarkers further increased AUC to 0.960 without considering the economic cost (Fig. 3C-D). Meanwhile, to optimize the diagnostic model for clinical application, we identified 5 variables (m^5^C modification, SCC, Cyfra21-1, CA125, and age) based on logistic regression analysis and constructed a nomogram model, which demonstrated excellent discrimination and reclassification performance in the diagnosis of NSCLC (Table 2; Fig. 5). Notably, our prediction model selected common clinical characteristics as predictors, making it ideal for replication in primary hospitals and areas with limited healthcare resources. As all the samples we used in this research were only collected from a single institution, further studies to verify the reliability of this model would require much larger sample sizes from other medical centers.

An accumulating body of research had indicated that RNA methylation modifications in peripheral blood cells were associated with disease diagnosis and prognosis. The concentrations of m^6^A in peripheral blood RNA had shown its potential as a biomarker to diagnose lung cancer, gastric cancer, and, breast cancer^12^, ^20^, ^21^. The demethyltransferase FTO has been found to exert a pro-carcinogenic effect in acute myeloid leukemia by regulating m^6^A levels on the mRNA of downstream target genes, such as ASB2 and RARA^22^. Additionally, regulated levels of m^6^A RNA modifications in peripheral blood following myocardial infarction might hold potential as a novel biomarker for the development of heart failure^23^. While previous studies had mainly focused on m^6^A in RNA methylation modification, little had been done on m^5^C. Our study found that the m^5^C modification was distinct from m^6^A modification in leukocytes, which was not abnormally high in all diseases.

To investigate the underlying reasons for the decreased m^5^C levels in leukocytes of NSCLC patients, we analyzed key regulatory factors previously implicated in m^5^C modification in NSCLC tumor tissues^15^. qRT-PCR analysis revealed a significant upregulation of the methylesterase NSUN3 and an apparent downregulation of the demethyltransferase TET2 in NSCLC patients compared to healthy individuals (Supplementary Figure. 1). However, no significant differences were observed in NOP2, ALYREF, and YBX1 (Supplementary Figure. 1). These findings suggested that NSUN3 and TET2 might contribute to the abnormal m^5^C modification levels in leukocytes of NSCLC patients, but further experimental data was needed to provide stronger evidence. Additionally, m^5^C modification leaded to the regulation of numerous downstream genes, and it remained crucial to investigate which specific leukocyte genes were affected by m^5^C modification and participated in tumor progression in NSCLC patients through further in vitro experiments.

With the development of molecular biology, liquid biopsy had become a hot research topic in tumor diagnosis^24^. Compared to traditional tissue biopsy, liquid biopsy was non-invasive and dynamic^25^. There were three main types of biomarkers of liquid biopsy for tumor patients: Circulating Tumor Cells (CTCs), Cell-Free DNA (cfDNA) and Exosomes (Exos). Epithelial-Mesenchymal Transition (EMT) was a biologic process that epithelial cells lost their adhesion and polarity to transform into mesenchymal cells, and CTCs were shed from the primary focus into the circulatory system^26^. CTCs were the first liquid biopsy indicator to be clinically tested, but tumor patients only contained 1-10 CTCs cells per mL of blood. Therefore, CTCs testing was more suitable for prognostic assessment of patients with advanced tumors, and its clinical application was limited^27^. Meanwhile, m^5^C modification could accurately distinguish early-stage NSCLC patients from healthy individuals with AUCs of 0.831 (Fig. 4). Continuous monitoring of cfDNA levels could also detect post-operative recurrence and metastases in gastric cancer patients up to 12 months earlier than imaging^28^. However, DNA fragments were also released during the death of normal cells and were more abundant, which might overwrite the cfDNA information of tumor cells and therefore were not suitable for early tumor diagnosis^29^. The method of isolation of Exos was not yet standardized, and different isolation methods could extract Exos with different characteristics, making them a long way from clinical application^30^, ^31^. In comparison, peripheral blood leukocyte samples were easy to obtain, and the m^5^C methylation detection method was inexpensive. While our research suggested that m^5^C methylation modification status of leukocytes held great promise as a superior diagnostic tool for NSCLC, there were certain challenges that must be addressed. For instance, storing RNA samples was difficult, and the process for detecting RNA methylation modifications was cumbersome.

While our findings hold promise, several limitations should be acknowledged in our study. Future investigations should aim to address these limitations, including the inclusion of larger sample sizes from multiple centers to mitigate potential biases such as NSCLC bias, control type bias, and selection bias. Additionally, long-term observation of lung cancer patients is warranted to determine whether m^5^C modification has a significant impact on prognosis. These efforts will provide a more comprehensive understanding of the implications of our findings and their clinical implications.

## Conclusion

In summary, our research indicated firstly the clinical diagnosis values of m^5^C methylation modification status in leukocytes of NSCLC. Additionally, combining m^5^C modification with other serum tumor biomarkers and constructing the nomogram model incorporating m^5^C modification could further enhance diagnostic efficacy.

## Supplementary Material

Supplementary figure and tables.

## Figures and Tables

**Table 1:** Sensitivity and specificity of the diagnostic value of various biomarkers alone and in combination in NSCLC.

Marker	Sensitivity	Specificity	AUC	95% CI
m^5^C	0.894	0.767	0.912	0.877-0.947
CEA	0.638	0.778	0.773	0.714-0.831
SCC	0.426	0.878	0.669	0.600-0.737
Cyfra21-1 CA125	0.504 0.509	0.989 0.867	0.754 0.732	0.692-0.815 0.665-0.800
Combination	0.851	0.956	0.960	0.938-0.982

**Table 2:** Univariate and multivariate logistic regression for NSCLC diagnosis.

Variables	Univariate Analysis			Multivariate Analysis		
	OR (95% CI)	*P* value		OR (95% CI)	*P* value	
m^5^C %						
<0.215 vs. ≥0.215	22.078 (21.340-22.815)	<0.001		26.310 (25.182-27.437)		<0.001
Gender						
Female vs. Male	1.080 (0.520-1.640)	0.788				
CEA (ng/mL)						
≥5 vs. <5	8.286 (7.533-9.039)	<0.001		2.775 (1.670-3.881)		0.070
SCC (ng/mL)						
≥1.5 vs. <1.5	7.280 (6.366-8.194)	<0.001		6.799 (5.176-8.423)		0.021
Cyfra21-1 (ng/mL)						
≥3.3 vs. <3.3	12.267 (11.510-13.024)	<0.001		10.701 (9.526-11.876)		<0.001
CA125 (ng/mL)						
≥35 vs. <35	20.343 (19.133-21.553)	<0.001		22.831 (21.181-24.480)		<0.001
Age	1.057(1.021-1.094)	0.003		1.120 (1.040-1.199)		0.006
						

## Abbreviations

ROC: Receiver operating characteristic
AUC: Area under the curve
NSCLC: Non-small cell lung cancer
m^5^C: 5-Methylcytosine
m^6^A: N6-methyladenosine
CEA: Carcinoembryonic antigen
SCC: squamous cell carcinoma antigen
Cyfra21-1: cytokeratin 19 fragment
CA125: carbohydrate antigen 125
CRC: colorectal cancer
CTCs: Circulating Tumor Cells
cfDNA: Cell-Free DNA
Exos: Exosomes
