# Supplementary material for: Diagnostic value of aberrant decreased 5-Methylcytosine RNA modification in leukocytes for non-small cell lung cancer
Source: J Cancer. 2023 Jul 16;14(12):2198–208. doi: 10.7150/jca.85681 (PMC10414042; doi:10.7150/jca.85681)
Supplement: Supplementary file 1 — Supplementary figure and tables. [file jcav14p2198s1.pdf]

Supplementary Table 1. Clinicopathological characteristics of the NSCLC patients and NC.

Characteristics	NSCLC (N=141)	NC (N=90)	*P* value
Age			
≤60	59(41.8%)	35(38.9%)	0.656
>60	82(58.2%)	55(61.1%)	
Gender			
Male	82(58.2%)	53(58.9%)	0.913
Female	59(41.8%)	37(41.1%)	
Clinical stage			
I	33(23.4%)	-	
II	23(16.3%)	-	
III	38(27.0%)	-	
IV	47(33.3%)	-	
T classification			
T1-T2	81(57.4%)	-	
T3-T4	60(42.6%)	-	
N classification			
N0	54(38.3%)	-	
N1-N3	87(61.7%)	-	
M classification			
M0	94(66.7%)	-	
M1	47(33.3%)	-	
Histopathological subtypes			
Adenocarcinoma	112(79.5%)	-	
Squamous cell carcinoma	24(17.0%)	-	
Large cell lung carcinoma	5(3.5%)	-	
Differentiation			
Poor	52(36.9%)	-	
Moderate / Well	85(60.3%)	-	
Missing	4(2.8%)	-	
EGFR genotyping			
Wildtype	22(15.6%)	-	
Mutation	21(14.9%)	-	
Missing	98(69.5%)	-	
TIF-1 expression			
Negative	34(24.1%)	-	
Positive	49(34.8%)	-	
Missing	58(41.1%)	-	
CEA (ng/mL)			
<5	71(50.4%)	84(93.3%)	<0.001
≥5	70(49.6%)	6(6.7%)	

SCC (ng/mL)			
<1.5	93(66.0%)	84(93.3%)	<0.001
≥1.5	48(34.0%)	6(6.7%)	
Cyfra21-1 (ng/mL)			
<3.3	57(40.4%)	80(88.9%)	<0.001
≥3.3	84(59.6%)	10(11.1%)	
CA125 (ng/mL)			
<35	60(42.6%)	88(97.8%)	<0.001
≥35	54(38.3%)	2(2.2%)	
Missing	27(19.1%)	-	

Supplementary Table 2. Correlation between m^{5}C modification and clinicopathological characteristics in NSCLC.

Characteristics	No.of patients	Peripheral blood m^{6}A levels % (mean±SD)	*P* value
Age			
≤60	59	0.123±0.067	0.248
>60	82	0.136±0.070	
Gender			
Male	82	0.116±0.059	0.004
Female	59	0.150±0.076	
Clinical stage			
I	33	0.177±0.061	<0.001
II	23	0.144±0.057	
III	38	0.110±0.062	
IV	47	0.108±0.069	
T classification			
T1-T2	81	0.147±0.062	0.076
T3-T4	60	0.124±0.070	
N classification			
N0	54	0.157±0.061	<0.001
N1-N3	87	0.114±0.068	
M classification			
M0	94	0.142±0.066	0.005
M1	47	0.108±0.069	
Histopathological subtypes			
Adenocarcinoma	112	0.134±0.072	
Squamous cell carcinoma	24	0.122±0.054	
Large cell lung carcinoma	5	0.092±0.047	
Differentiation			
Poor	52	0.115±0.069	0.041
Moderate / Well	85	0.139±0.068	
Missing	4	0.133±0.050	
EGFR genotyping			

Wildtype	22	0.166±0.066	0.016
Mutation	21	0.114±0.069	
Missing	98	0.126±0.067	
TIF-1 expression			
Negative	34	0.128±0.072	0.212
Positive	49	0.148±0.073	
Missing	58	0.117±0.060	
CEA (ng/mL)			
<5	71	0.143±0.066	0.024
≥5	70	0.117±0.070	
SCC (ng/mL)			
<1.5	93	0.138±0.070	0.069
≥1.5	48	0.116±0.064	
Cyfra21-1(ng/mL)			
<3.3	57	0.144±0.064	0.049
≥3.3	84	0.121±0.071	
CA125 (ng/mL)			
<35	60	0.129±0.073	0.828
≥35	54	0.132±0.066	
Missing	27	0.126±0.057	

Supplementary Table 3. Sensitivity and specificity of the diagnostic value of various biomarkers in different stage of NSCLC.

Stage	Marker	Sensitivity	Specificity	AUC	95% CI
I	m^5C	0.879	0.678	0.831	0.756-0.905
	CEA	0.667	0.511	0.594	0.472-0.715
	SCC	0.485	0.700	0.563	0.440-0.686
	Cyfra21-1	0.455	0.889	0.644	0.521-0.767
	CA125	0.520	0.722	0.627	0.492-0.763
II	m^5C	0.957	0.800	0.912	0.855-0.968
	CEA	0.739	0.622	0.713	0.578-0.848
	SCC	0.565	0.844	0.717	0.591-0.844
	Cyfra21-1	0.696	0.800	0.728	0.579-0.877
	CA125	0.471	0.967	0.724	0.559-0.888
III	m^5C	0.895	0.911	0.950	0.909-0.991
	CEA	0.816	0.833	0.894	0.834-0.955
	SCC	0.605	0.878	0.776	0.676-0.876
	Cyfra21-1	0.737	0.822	0.807	0.712-0.903
	CA125	0.424	1.000	0.750	0.645-0.854
IV	m^5C	0.851	0.922	0.939	0.897-0.981
	CEA	0.617	0.933	0.849	0.779-0.919
	SCC	0.596	0.933	0.782	0.689-0.875
	Cyfra21-1	0.660	0.989	0.800	0.700-0.899
	CA125	0.462	0.967	0.769	0.677-0.861

Supplementary Figure 1. Expressions of m5C-modified regulators in leukocytes of NSCLC patients.

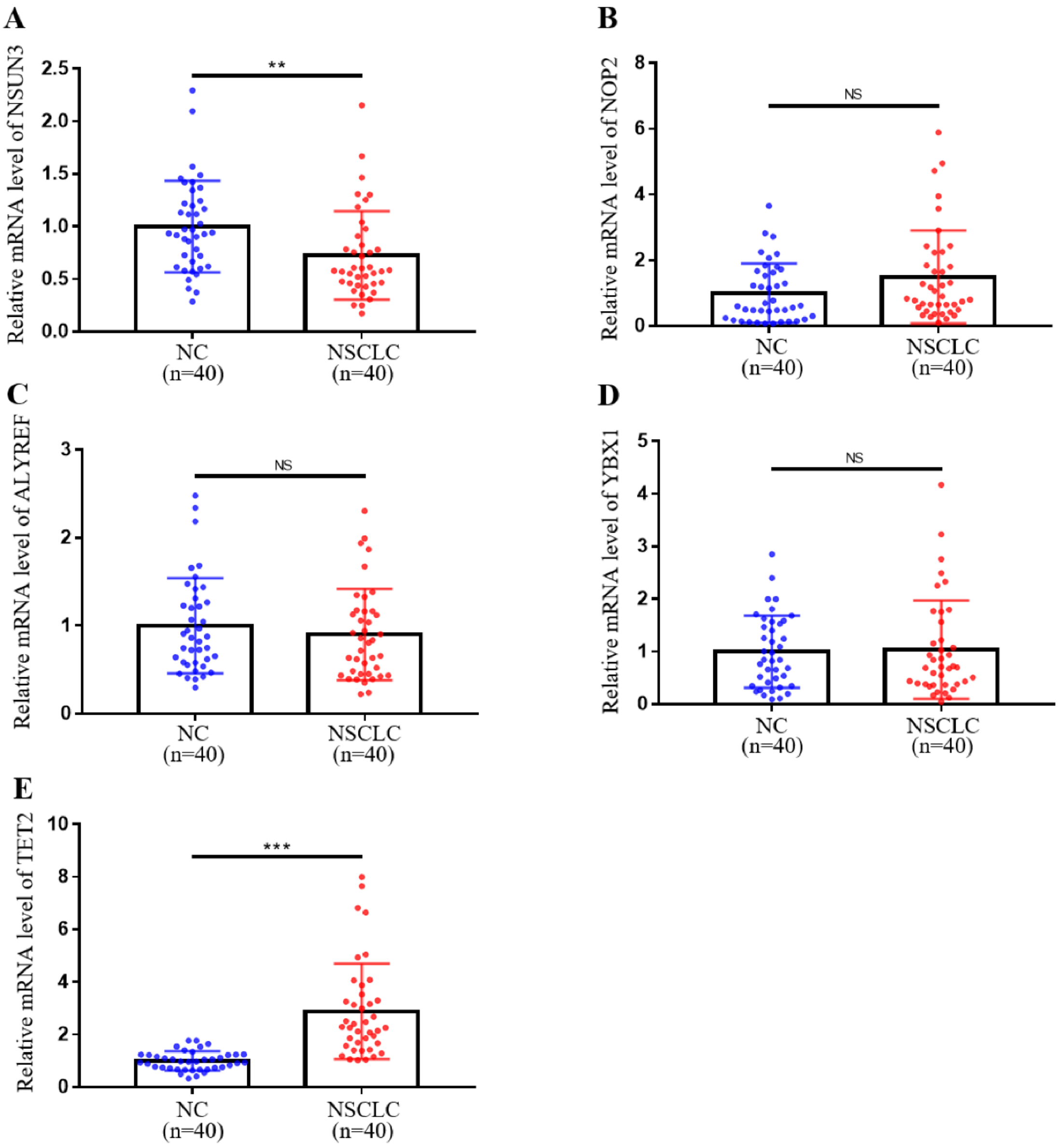

(A-E) qRT-PCR analysis of NSUN3 (A), NOP2 (B), ALYREF (C), YBX1 (D), and TET2(E) mRNA expression levels in leukocytes of NC and NSCLC patients.
